# Sample Entropy on Multidistance Signal Level Difference for Epileptic EEG Classification

**DOI:** 10.1155/2018/8463256

**Published:** 2018-09-12

**Authors:** Achmad Rizal, Sugondo Hadiyoso

**Affiliations:** ^1^School of Electrical Engineering, Telkom University, Bandung 40257, Indonesia; ^2^Telkom Applied Science School, Telkom University, Bandung 40257, Indonesia

## Abstract

Epilepsy is a disorder of the brain's nerves as a result of excessive brain cell activity. It is generally characterized by the recurrent unprovoked seizures. This neurological abnormality can be detected and evaluated using Electroencephalogram (EEG) signal. Many algorithms have been applied to achieve high performance for the EEG classification of epileptic. However, the complexity and randomness of EEG signals become a challenge to researchers in applying the appropriate algorithms. In this research, sample entropy on Multidistance Signal Level Difference (MSLD) was applied to obtain the characteristic of EEG signals, especially towards the epilepsy patients. The test was performed on three classes of EEG data: EEG signals of epilepsy patient in ictal (seizure), interictal conditions (occurring between seizures) and normal EEG signals from healthy subjects with a closed eye condition. In this study, classification and verification were done using the Support Vector Machine (SVM) method. Through the 5-fold cross-validation, experimental results showed the highest accuracy of 97.7%.

## 1. Introduction

Biological signals are the complex signals resulting from some complex physiological processes in the body [1]. Complex signals are signals that have some properties between periodic signals and random signals. These signals are analyzed using several points of view, such as fractal, entropy, or chaotic approaches. One commonly used method for complex signal analysis is multiscale entropy (MSE). Costa et al. proposed MSE method for a biological signal analysis [2]. As the biological signals are considered to have a number of multiscale properties, an analysis on multiple scales will provide the complete signal characteristic information.

For many cases, one of the most commonly biological signals analysis using MSE is the Electroencephalogram signal (EEG). The measurements of brain functions through EEG can be used for monitoring and interpreting the brain activity, even predicting the outcomes [3]. MSE was used for the analysis of EEG signals monitoring the depth of the anesthetic process during surgery [4]. The results showed that MSE at the presurgical stage was lower than the one at the anesthesia stage. MSE is also used to measure the dynamics of EEG signal complexity in patients with Alzheimer's disease (AD) [5]. Although statistically the difference between normal EEG and AD is not very significant, there is a difference in pattern between MSE in EEG signals in the normal AD and EEG patients. Lu et al. used MSE in EEG signals as a predictor for the prognosis of neonatal seizures [6]. The EEG signal was acquired from 32 infants below two months old and analyzed using sample entropy, multiscale entropy, and complexity index (CI). The value of MSE and CI decreased in infants who experienced seizures. Attention-related EEG based on motor imaginary potential using multiscale entropy analysis was reported in [7]. MSE was used to differentiate EEG signals recorded in three attention-related activities and obtained the accuracy of 63.158%. Other research [8, 9] performed an analysis of epileptic EEG signals compared with the normal subjects' EEG signals using the Detrended Cross-Correlation Analysis (DCCA) method. From this research, it can be concluded that the DCCA value of epileptic EEG signal was greater than normal subjects' EEG signals. A review paper on the application of entropies methods on recognition of epilepsy using EEG signals was presented in [10]. The paper presented a comparison of various entropy methods used for the classification of normal, interictal, and ictal EEG signals. Many researchers have experimented with different entropy in the analysis and classification of EEG signals. Some have used one entropy feature, and others have used the combination of entropies. Both experiments have been reported to reach the accuracy of more than 92%. From this review, it can be concluded that entropy is one of the state-of-the-art methods that have a good performance for recognizing EEG signals in normal, ictal, and interictal conditions, which may be difficult to be recognized visually. Entropy can also be used in focal cases and nonfocal EEG signals.

From the related works described above, the method of feature extraction plays an essential role in the pattern recognition, especially EEG signals. In this research, we have simulated and analyzed the sample entropy (SampEn) on Multidistance Signal Level Difference (MSLD) for feature extraction and SVM algorithm for epileptic EEG signal classification. MSLD was selected for having good performance on the results of a previous study [11]. The MSLD segmented the EEG and then calculated the SampEn at each of its MSLD levels. Then, for classification, we used the Support Vector Machine (SVM) method. The test was performed on three EEG data classes; those are EEG signals of epilepsy patient in ictal conditions, EEG signals in interictal conditions, and normal EEG signals from healthy subjects. All datasets used in this study were sourced from the open databases available at the University of Bonn. The data was taken from normal subjects and epileptic subjects with interictal and ictal conditions.

## 2. Materials and Methods

### 2.1. EEG Data

**Figure 1:**
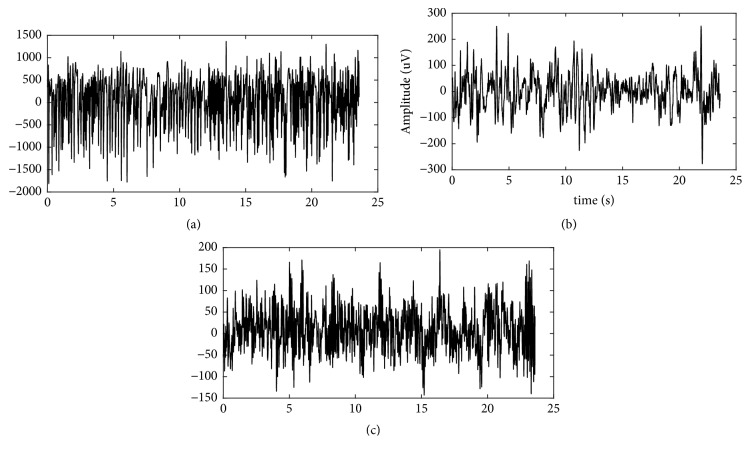
(a) ictal EEG, (b) interictal EEG, (c) normal EEG.

In this research, we used the EEG dataset available at the University of Bonn [12] (source: http://epileptologie-bonn.de/cms/upload/workgroup/lehnertz/eegdata.html). Data were recorded using 173.61 Hz sampling frequency and filtered using 40 Hz LPF. Thus, it was free from artifacts noise. Each data had a length of 4096 samples with the duration of 23.6s. In this study, we used three classes of EEG data consisting of EEG signals from epileptic subjects in the condition of seizures (ictal), EEG signals in interictal conditions, and normal EEG signals from a healthy person with a closed eye condition. Ictal and interictal data were obtained from five patients with pharmacoresistant focal onset epilepsy undergoing some presurgical evaluations. These patients had the long-term intracranial EEG recording in the Department of Neurology, University of Bern. Some electrodes were implanted on the brain area to record the interictal segments between seizures or conditions at intervals without seizures. Each data class consists of 100 dataset; thus, a total of 300 EEG datasets were tested in this study. The sample data for each class can be seen in Figure 1.

### 2.2. Multidistance Signal Level Difference

Multidistance Signal Level Difference (MSLD) is a modification of the gray-level difference (GLD) proposed by Weszka et al. [13]. GLD was calculated from the absolute value of the difference of two adjacent pixels in the horizontal, vertical, and diagonal directions [11]. In the horizontal direction, GLD could be calculated as

(1) $$\begin{array}{c} y\left(\;i,j\;\right)=\left|\;x\left(\;i,j\;\right)-x\left(\;i,j+D\;\right)\;\right|, \end{array}$$

where D is the pixel distance.

**Figure 2:**
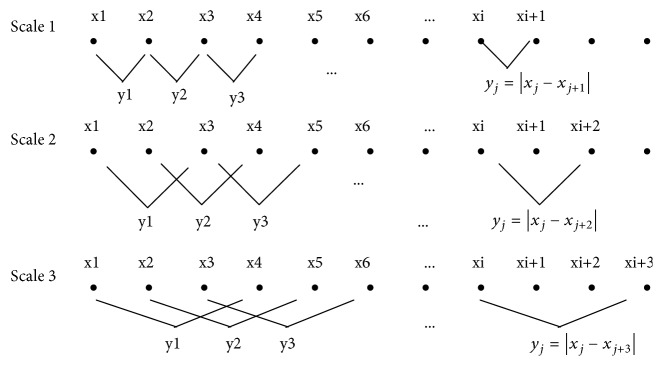
Illustration of MSLD [11].

In MSLD, since the signal used was 1D (one dimension), then (1) was modified to (2). The illustration of MSLD in the diagram can be seen in Figure 2.

(2) $$\begin{array}{c} y_{d}\left(\;i\;\right)=\left|\;x\left(\;i\;\right)-x\left(\;i+d\;\right)\;\right|, \end{array}$$

where *i* = 1,2,…, *N* − *d* and *d* = 1,2, .., *K*.

### 2.3. Sample Entropy

Sample entropy (SampEn) was proposed by Richman and Moorman to resolve the weakness of ApEn [14]. In ApEn, there was a bias due to self-matches where the code template of the signal was considered equal to itself. SampEn is the probability of the *m* sequence of data that will be the same as other sequences in the sequence of signals with the tolerance r, which will remain the same if the sequence m of data is increased to m + 1. Equally, in this case, it has a scale distance between 2 vectors compared to [15]. The equation of SampEn is expressed by

(3) $$\begin{array}{c} SampEn\left(\;m,r\;\right)=\underset{N\to \infty }{\lim}-\ln\frac{A^{m}\left(r\right)}{B^{m}\left(r\right)} \end{array}$$

where *A*^*m*^(*r*) is the probability of two data sequences that would match for a number m+1 point in tolerance r. Meanwhile, *B*^*m*^(*r*) is the probability of two data sequences that would match for a number m point in tolerance r. In both parameters, self-matches have been avoided. Furthermore, (3) can be estimated by

(4) $$\begin{array}{c} B=\left\{\;\frac{\left[\left(N-m-1\right)\left(N-m\right)\right]}{2}\;\right\}B^{m}\left(\;r\;\right) \end{array}$$

and

(5) $$\begin{array}{c} A=\left\{\;\frac{\left[\left(N-m-1\right)\left(N-m\right)\right]}{2}\;\right\}A^{m}\left(\;r\;\right). \end{array}$$

Then, SampEn can be expressed by

(6) $$\begin{array}{c} SampEn\left(\;m,r,N\;\right)=-\ln\frac{A}{B}. \end{array}$$

The advantages of SampEn include its usability for short data sequence with noise, its ability to separate large system variations, its better performance compared to ApEn according to theory, its consistent entropy values for different pattern lengths, and self-matches not calculated. The weakness of SampEn is related to the inconsistency of entropy values for short data [10].

### 2.4. Support Vector Machine

Support Vector Machine (SVM) is one of the machine learning algorithms widely used for pattern recognition. The method proposed by [16] is principled on Structural Risk Minimization (SRM) with the aim of finding the best hyperplane to separate two classes in a space. SVM is a popular algorithm with high performance that is widely used for classification in cases that have complex computations [17].

**Figure 3:**
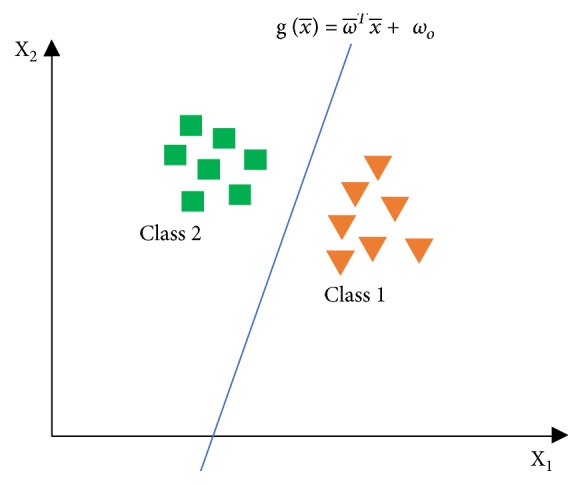
Hyperplane classifies data into two classes.

**Figure 4:**
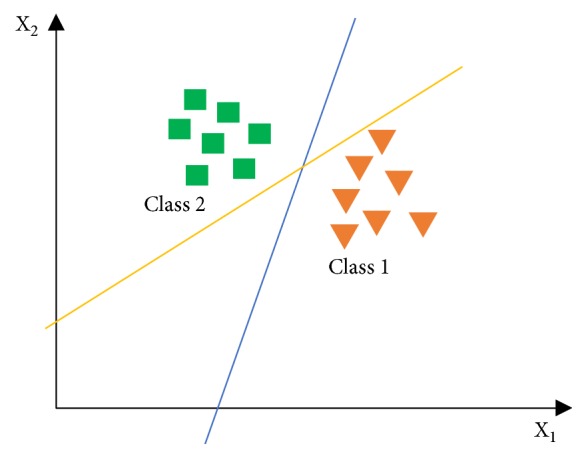
Illustration of finding the best hyperplane between two classes.

SVM primarily works on linear problems and then it is developed to be used on nonlinear problems. It works on the kernel trick concepts in high-dimensional workspaces. The SVM concept is to design a hyperplane that can classify all training data into two classes.  Figure 3 shows some patterns that are the members of two classes in the form of triangles and squares. Various alternate lines of discrimination (discrimination boundaries) are shown in Figure 4.

SVM works by applying the kernel function to form two classes in the training data. Commonly, there are three kinds of kernel functions that can be used. The first type is linear kernel function with the equation below:

(7) $$\begin{array}{c} K\left(\;X,Y\;\right)=X^{T}Y. \end{array}$$

The second kind is the polynomial kernel function:

(8) $$\begin{array}{c} k\left(\;X_{i},X_{j}\;\right)={\left(\;X_{i},X_{j}\;\right)}^{d} \end{array}$$

where d (d ≥1) is the number of polynomials. If d = 2 or d = 3, the function is defined as a quadratic kernel or cubic kernel function.

### 2.5. K-Fold Cross-Validation

**Figure 5:**
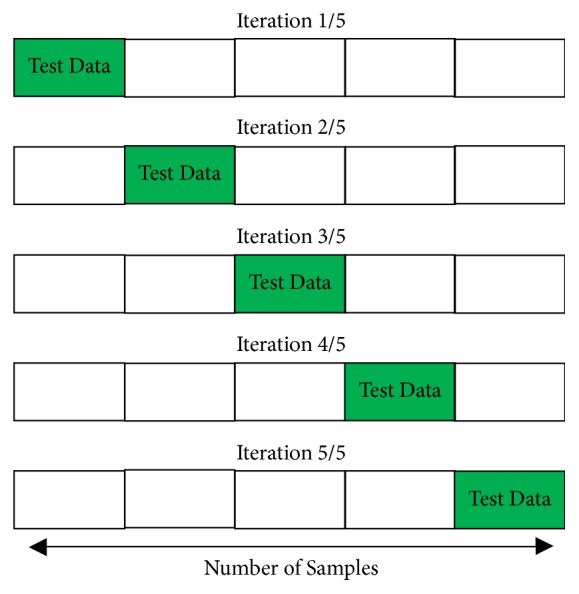
Illustration of 5-fold CV.

Performing the validity testing of machine learning algorithm requires the performance evaluation through cross-validation. Here, the dataset would be separated into two subsets consisting of training data and test data. In K-Fold cross-validation, the data is divided into k subsets. In this research, we conducted a 5-fold CV simulation. In 5-fold CV, data was divided into 5-fold groups, enabling us to have five subsets of data. From 5 subsets of data, there were four subsets of training data and one subset of test data. Thus, it would be iterated five times as illustrated in Figure 5.

## 3. Results and Discussion

**Figure 6:**
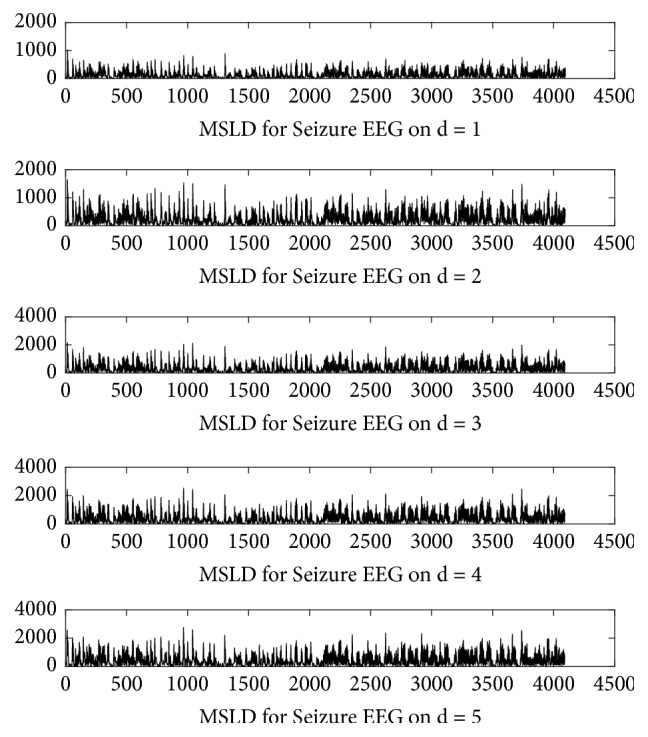
MSLD results of seizure EEG signal.

The result of the MSLD process for the seizure EEG signal with distance d=1-5 is shown in Figure 6. MSLD calculated the absolute value of the difference of 2 data samples at distance *d* so that the resulting signal was always in the form of a positive value. New signals generated by MSLD would have a number of properties slightly different from the original signal, and these features would be quantized using sample entropy.

**Figure 7:**
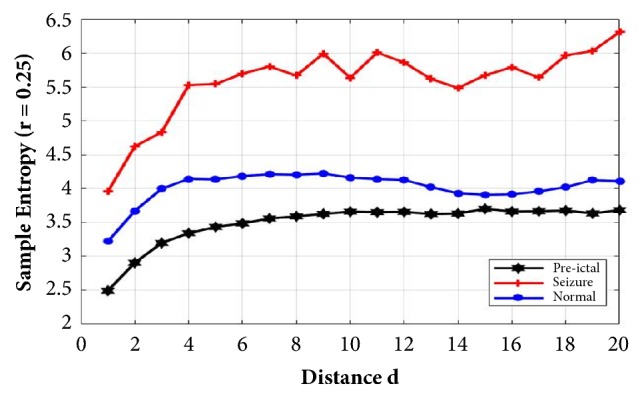
Sample entropy (r = 2.5) with MLSD for each distance d.

Sample entropy value for each data class of MSLD results can be seen in Figure 7. Sample entropy was calculated with r = 0.25. It can be seen that EEG seizure produced the highest SampEn value compared to other conditions and the interictal conditions produced the lowest one. This proved that the condition of the seizure of EEG signal had the highest complexity value. Visually, the value of SampEn between the three classes was significantly different so that in the classification process it could be differentiated well.

The next process was the performance testing of MSLD-SampEn using SVM with multiple kernels as a classifier. The test was performed using 20 SampEn values prior to subtracting the features used to see the effect of feature reduction on accuracy. The results are shown in Tables 1, 2 and 3.

From Tables 1, 2, and 3, the highest accuracy is 97.7% with the use of cubic SVM, MSLD with distance d=1 -20, and SampEn with r=0.25. It can then be seen that reducing the number of features could decrease the resulting accuracy, except for r = 0.25 using linear SVM and r = 0.1 using cubic SVM.

MSLD shows a common occurrence of two samples at a specific distance range. These results showed some differences of features between classes. The advantage of MSLD is that the value of signal variance is unchanged, different from coarse-grained procedures that decrease the value of signal variance as discussed in previous studies [11]. A decrease in signal variance indicates a change of signal feature so that the results of the coarse-grained procedure will change the feature of the original signal. In previous research of lung sound classification, MSLD Hjorth descriptor obtained higher accuracy than multiscale Hjorth descriptor using coarse-grained procedure [18]. The disadvantage of MSLD is that the range of distances to be calculated is determined by trial and error. However, empirically the MSLD is well calculated for the range of distance d=1-15.

The MSLD method can be further developed in combination with other various feature extraction methods such as other entropy computation, statistical, or signal complexity methods. MSLD can also be used to manipulate some biological signals in addition to EEG signals or lung sounds [11]. MSLD method for signal classification such as ECG, EMG, or other biological signals will be interesting research in the future.

## 4. Conclusion

This research describes the classification of epileptic EEG signals using MSLD sample entropy. Tests were performed on three classes of EEG signals: normal, seizure, and interictal. This dataset is available online from Department of Epileptology, University of Bonn. From the feature extraction process, the sample of entropy for each class with r=0.25 showed a different value. In the EEG seizure signal, it produced the highest SampEn value compared to the other two conditions. From the simulation results, the values of sample entropy for each class could be differentiated enabling it to be easily classified. We also tested the classifier performance by applying SVM to the MSLD-SampEn result. The test results showed the highest accuracy of 97.7% using the MSLD with distance d=1 -20, SampEn with r = 0.25, and cubic SVM. MSLD can be well used to search some differences in sample signals with apparent difference values. For further research, it is suggested that MSLD can be used for the classification of other biopotential signals that have high complexity.

## Figures and Tables

**Table 1:** Accuracy (%) using linear SVM and 5-fold CV.

SampEn	Scale 1-20	Scale 1-15	Scale 1-10	Scale 1-5
r = 0.1	96	95.7	84.3	79.7
r = 0.15	96	95	83.7	78.7
r = 0.2	96	96	84	78.7
r = 0.25	95.7	96	84	78

**Table 2:** Accuracy (%) using quadratic SVM and 5-fold CV.

SampEn	Scale 1-20	Scale 1-15	Scale 1-10	Scale 1-5
r = 0.1	96.7	95.7	85.7	81.7
r = 0.15	95.3	96.7	86.3	82.7
r = 0.2	96.7	95.7	87.3	82.7
r = 0.25	97.7	96.7	85.3	80.7

**Table 3:** Accuracy (%) using cubic SVM and 5-fold CV.

SampEn	Scale 1-20	Scale 1-15	Scale 1-10	Scale 1-5
r = 0.1	96.3	97	85.7	85
r = 0.15	97	89.7	84.3	73.3
r = 0.2	97	96	88.3	82
r = 0.25	97.7	95.7	83.7	83

## Data Availability

The data used to support the findings of this study are available from open databases (http://epileptologie-bonn.de/cms/upload/workgroup/lehnertz/eegdata.html).
